# Alterations of Plasma Lysophosphatidylcholine Species in Obesity and Weight Loss

**DOI:** 10.1371/journal.pone.0111348

**Published:** 2014-10-23

**Authors:** Susanne Heimerl, Marcus Fischer, Andrea Baessler, Gerhard Liebisch, Alexander Sigruener, Stefan Wallner, Gerd Schmitz

**Affiliations:** 1 Institute of Clinical Chemistry and Laboratory Medicine, University Medical Centre, Regensburg, Germany; 2 Clinic for Internal Medicine II, University Medical Centre, Regensburg, Germany; National Institute of Nutrition, India

## Abstract

**Background:**

Obesity and related diseases of the metabolic syndrome contribute to the major health problems in industrialized countries. Alterations in the metabolism of lipid classes and lipid species may significantly be involved in these metabolic overload diseases. However, little is known about specific lipid species in this syndrome and existing data are contradictive.

**Methods:**

In this study, we quantified plasma lipid species by electrospray ionization tandem mass spectrometry (ESI-MS/MS) in obese subjects before and after 3 month weight loss as well as in a control group.

**Results:**

The comparison of obese subjects with control subjects before weight loss revealed significantly lower lysophosphatidylcholine (LPC) concentrations in obesity. LPC concentrations did not significantly increase during the observed period in the weight loss group. Analysis of LPC species revealed a decrease of most species in obesity and negative correlations with C-reactive protein (CRP) and body mass index (BMI). Correlating BMI ratio before and after weight loss with the ratio of total LPC and individual LPC species revealed significant negative relationships of LPC ratios with BMI ratio.

**Conclusions:**

Our findings contribute to the contradictive discussion of the role of LPC in obesity and related chronic inflammation strongly supporting pre-existing data in the literature that show a decrease of LPC species in plasma of obese and a potentially anti-inflammatory role in these subjects.

## Introduction

Obesity has become one of the major worldwide health problems. Obesity is associated with a variety of severe diseases and co-morbidities including type 2 diabetes, atherosclerosis, and hypertension. These health problems are well-known to be related to alterations in plasma lipids like increased fasting triglycerides, high low density lipoprotein (LDL) cholesterol, low high density lipoprotein (HDL) cholesterol and elevated blood glucose and insulin levels [1]. However, only very few and in part, contradictive data exist on alterations of the plasma lipidome in obesity and subsequent weight loss.

Mass spectrometry-based lipid analysis today allows profiling of a large variety of lipid classes and species in a single sample. This technology has been successfully applied in studies of metabolic disorders. In particular, in type 2 diabetes (T2D) specific plasma phospholipids have been associated with insulin sensitivity [2], the inclusion of plasma lipid species significantly improved the classification of individuals at risk for T2D [3]. T2D and prediabetes were positively associated with plasma ceramide (Cer), dihydroceramide, phosphatidylethanolamine (PE), phosphatidylglycerol and phosphatidylinositol (PI) while a negative relationship has been shown with ether-linked phosphatidylcholines (PC) [4]. Moreover, in coronary artery disease, several lipid species including alkylphospholipid and PI species were identified as discriminatory for stable and unstable coronary artery disease [5]. In hypertensive patients a deficiency in plasma ether lipids, specifically in ether PC and ether PE, has been demonstrated [6].

Since obesity is strongly related to these diseases it seems obvious that relevant alterations of the plasma lipidome may be observed in these subjects. A marked increase in plasma levels of saturated di- and triacyglycerols as well as increased levels of PC, PE, PE ether and lysophosphatidylcholine (LPC) have been reported in obesity [6]. In a monozygotic twin study obesity was primarily related to increases in LPC and to decreases in ether phospholipids [7]. In contrast, Barber et al. [8] found in a small cohort a reduction in a number of LPC species in obese and obese individuals with T2D.

In a study of diet-induced weight loss in obese, a reduction of PC and, predominantly short chain fatty acid triacylglycerols in serum was observed, while other lipid classes as sphingolipids and LPC remained unaffected by weight loss [9].

Interestingly, alterations of plasma LPC are found in most studies concerned with obesity. Plasma LPC mainly originates from lecithin-cholesterol acyltransferase (LCAT) activity [10], hepatic secretion [11] or from PC by action of phospholipase A_2_ [12].

To further elucidate these contradictive data on plasma lipid classes and species in obesity and their potential role in the development of metabolic abnormalities, we here investigated obese subjects before and three months of diet-induced weight loss and compared them to lean controls. Using an ESI-MS/MS based lipidomic approach, we performed quantitative lipidomic profiling as well as routine clinical chemistry of individual plasma samples before and after weight loss.

## Methods

### Study population

The study population of the ongoing “Obesity and Weight Reduction Remodeling Study” has been described previously [13], [14]. Control and obese subjects for the study population published here have been recruited from 03/2005 to 04/2008 at the University Hospital of Regensburg.

Obese patients intending to participate in a weight reduction programme were offered enrolment in this study, prior to the start of the programme. Patients were eligible for enrolment, if they were 18–59 years old, presented with a body mass index (BMI)>30 kg/m^2^ and a constant body weight in the last 3 months, and if they signed a declaration of consent. Patients were excluded, if they had one or more of the following:>10% reduction of body weight in the last 6 months, cancer, pregnancy, therapy with steroids or thyroid hormones, known heart disease, known type 1 or type 2 diabetes, known inflammatory bowel, rheumatoid, or systemic diseases, known chronic renal failure, known liver diseases, mental disorders, or addiction to drugs or alcohol.

For comparison, healthy normal weight control subjects (BMI 20–25 kg/m^2^) of comparable age and gender distribution were also studied. They were recruited by flyers, advertisements, and friend referrals. The study was approved by the local Ethics Committee (Universitätsklinikum Regensburg, Ethikkommission der medizinischen Fakultät, proposal 05/001). All subjects had given their written informed consent to participate in the study.

The baseline characteristics of the study population are summarized in ***Table 1***.

**Table 1:** Baseline characteristics in obese and control subjects before and after weight loss.

	Controls		obese	
	Before weight loss	After weight loss	Before weight loss	After weight loss
N (all)	23		57	
N(male)	9		25	
N (female)	14		32	
Age (years)	38.22±13.77		46.40±13.14*	
BMI (kg/m2)	22.55±2.13	22.51±2.17	41.62±8.18***	35.00±7.74^###;•••^
Waist (cm)	78.95±7.72	77.26±5.90	124.32±17.90***	110.27±17.67^###.^ •••
Hip (cm)	99.95±4.56	98.67±4.29	133.21±15.88***	121.73±14.87^###.^ •••
Waist/Hip	0.79±0.06	0.78±0.05	0.94±0.09	0.91±0.09^###.^ ••
Chloesterol (mg/dl)	187.57±25.18	196.23±30.76	192.46 ±39.80	169.04±32.38^##.^ •••
LDL cholesterol (mg/dl)	105.05± 25.18	113.39±28.98	117.04±38.07	99.82±31.42•••
HDL cholesterol (mg/dl)	64.76±9.79	64.83±11.77	48.93±12.09***	45.88±10.03^###.^ •
Triglyceride (mg/dl)	68.86±24.95	90.26±38.79	140.09±77.02***	100.98±40.64•••

*p<0.05 obese vs. control before weight loss.

*** p<0.001 obese vs. control before weight loss.

## p<0.01 obese vs. control after weight loss.

### p<0.001 obese vs. control after weight loss.

• p<0.05 obese before vs. after weight loss.

•• p<0.01 obese before vs. after weight loss.

••• p<0.001 obese before vs. after weight loss.

### Weight reduction program

Weight reduction in obese subjects was pursued by Optifast-52 (Nestlé HealthCare Nutrition GmbH) comprising a 52-week medical weight loss program encompassing diet, lifestyle changes, counseling and exercises. For the present study, only data of the initial three month of the “Active Weight Loss Phase” were utilized. During this phase, patients consume 800 kilocalories per day consisting only of meal replacements supplied by Optifast (shakes, nutritional bars, soups) and water.

### Phenotyping and plasma sampling

Blood samples were drawn after a 12-h overnight fasting between 7:00 and 9:00 a.m. before starting (t = 0) and after three months of the weight reduction program (t = 1). At the same time, height and weight were recorded. Serum samples for lipid analysis were analyzed the same day, for lipid species analysis, EDTA-pasma samples were stored at −80°C before measurement.

### Clinical chemistry and lipid analysis

Cholesterol, HDL-cholesterol, LDL-cholesterol and triglycerides were determined on ADVIA 1800 System (Siemens), using commercial kits.

Lipids were quantified by direct flow injection electrospray ionization tandem mass spectrometry (ESI-MS/MS) in positive ion mode, using the analytical setup and strategy described previously [15], [16]. A precursor ion of *m/z* 184 was used for PC, sphingomyelin (SM) [15] and LPC [17]. Neutral loss fragments were used for the following lipid classes: PE and PI with a loss of 141 and 277 Da, respectively [18], [19]. PE-based plasmalogens (PE-P) were analyzed according to the principles described by Zemski-Berry [20]. Sphingosine-based Cer and hexosylceramides (HexCer) were analyzed, using a fragment ion of *m/z* 264 [21]. Free cholesterol (FC) and cholesteryl ester (CE) were quantified, using a fragment ion of *m/z* 369, after selective derivatization of FC [16]. Quantification was acchieved using two non-naturally occurring internal standards (IS) for each lipid class (except for PI, SM was calculated, using PC IS and PE-based plasmalogens were calculated, using PE IS) and calibration lines generated by standard addition of a number of naturally occurring species to plasma. Deisotoping and data analysis for all lipid classes was performed by self programmed Excel Macros as described previously [15], [22]. Lipid species were annotated, according to the recently published proposal for shorthand notation of lipid structures that are derived from mass spectrometry [23]. Glycerophospholipid species annotation was based on the assumption of even numbered carbon chains only. SM species annotation is based on the assumption that a sphingoid base with two hydroxyl groups is present.

### Statistical analysis

All data are expressed as mean ± SD. Statistical analysis was performed using IBM SPSS statistics 19 software. Statistical significance was determined by the Mann-Whitney-U-test for comparing controls vs. obese and the Wilcoxon-paired-samples-test for comparing subjects before and after weight loss. Linear relationships were determined by the Pearson correlation coefficient. A p<0.05 was considered as statistically significant.

## Results

### Plasma lipid profile in obesity and weight loss

The lipid species of each class were examined by measuring individual samples. As expected, CE was the most abundant lipid in control samples (3991±576 µM). Other major lipid classes were PC (1806±303 µM), SM (441±64 µM), and LPC (287±66 µM). All other analyzed lipid classes were detected at levels lower than 50 µM.

**Figure 1:**
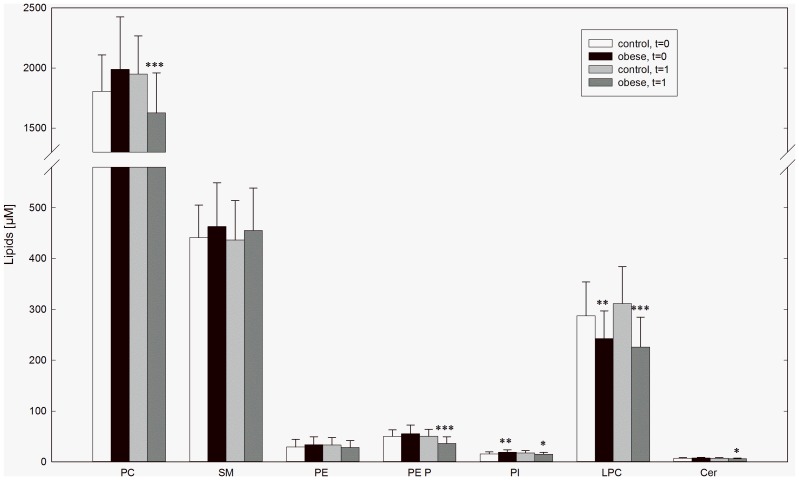
Plasma levels of lipid classes in obese and control subjects. Lipid concentrations in EDTA-plasma were measured by ESI-MS/MS in obese and control subjects at entry in the study (t = 0) and after 3 month of weight loss of obese subjects (t = 1). Data are expressed as mean ± SD. *p<0.05 obese vs. control, **p<0.01 obese vs. control *** p<0.001 obese vs. control.

The comparison of obese subjects with control subjects before weight loss (t = 0) revealed significantly lower LPC concentrations in obesity (control vs. obese: 287±66 µM vs. 243±54 µM, p = 0.005). In addition FC (1176±175 µM vs. 1309±266 µM, p = 0.039), PI (15.6±4.0 µM vs. 18.9±4.9 µM, p = 0.032), dihydrosphingomyelin (8.0±2.6 µM vs. 12.1±3.9 µM, p<0.001) were elevated. After 3 month of weight loss (t = 1) hexosylceramide (0.8±0.1 µM vs. 1.0±0.3 µM, p = 0.007) and dihydrosphingomyelin (7.8±2.4 µM vs. 10.9±4.0 µM, p<0.001) were elevated in obese subjects compared to controls. Lower LPC concentrations in obesity could still be observed (311±73 µM vs. 226±59 µM, p<0.001) at t = 1. In obese lower levels of total cholesterol (4023±539 µM vs. 3587±652 µM, p = 0.005), PC (1951±316 µM vs. 1626±333 µM, p<0.001), plasmalogens (50.3±13.8 µM vs. 36.1±13.0 µM, p<0.001), PI (17.7±4.3 µM vs. 15.2±3.4 µM, p = 0.032), and Cer (7.0±1.2 µM vs. 6.2±1.2 µM, p = 0.027) were observed after weight loss. *(* ***Figure 1*** *)*.

The observed effects were independent of sex. In particular, the observed differences between lean and obese subjects in plasma LPC remained significant in a gender specific evaluation. LPC was lower in plasma of males (307±60 µM vs. 249±66 µM, p = 0.037) and females (275±69 µM vs. 238±44 µM, p = 0.048) before weight loss, and lower levels were also observed in males (351±78 µM vs. 241 µM±67 µM, p<0.001) and females (286±58 µM vs. 214±50 µM, p<0.001) after 3 month weight loss.

### Plasma LPC species in obesity and weight loss

**Figure 2:**
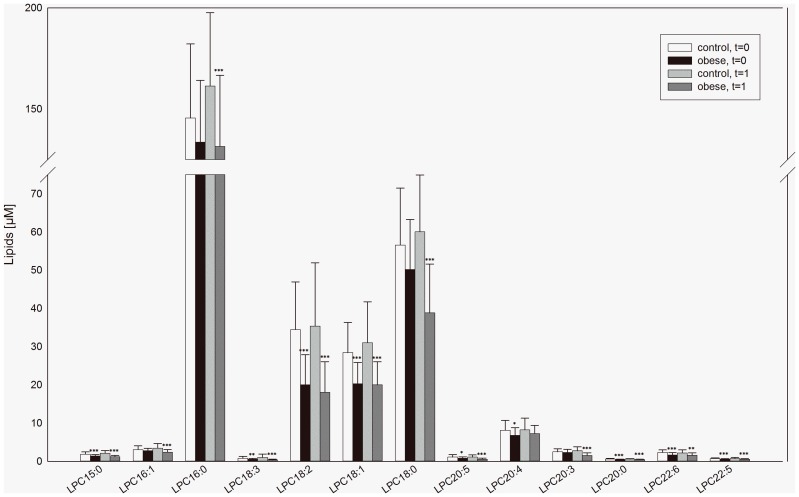
Plasma lysophosphatidylcholine levels in obese and control subjects. Lysophosphatidycholine (LPC) species in EDTA-plasma were measured by ESI-MS/MS in obese and control subjects at entry in the study (t = 0) and after 3 month of weight loss of obese subjects (t = 1). Data are expressed as mean ± SD. *p<0.05 obese vs. control, **p<0.01 obese vs. control *** p<0.001 obese vs. control.

To elucidate whether the decrease in plasma LPC in obesity is due to alterations in certain LPC species, the concentration of distinct LPC species was analyzed in all samples included in the study. LPC 16∶0 was the most abundant species in control subjects (146±37 µM) followed by LPC 18∶0 (56.5±14.9 µM), LPC 18∶2 (34.5±12.5 µM) and LPC 18∶1 (28.4±12.5 µM). All other LPC species were detected at concentrations below 10 µM. At t = 0 in obese subjects significantly lower plasma concentrations were found in almost all LPC species analyzed, namely LPC15∶0, LPC 18∶3, LPC 18∶2, LPC 18∶1, LPC 20∶5, LPC 20∶4, LPC 20∶0, LPC 22∶6, and LPC 22:5. After 3 month weight loss (t = 1), besides for LPC 20∶4 the observed differences in plasma levels persisted. Moreover, in addition to the LPC species mentioned above LPC 16∶1, LPC16∶0, LPC 18∶0 and LPC 20∶3 were found decreased in obese subjects at t = 1. *(* ***Figure 2*** *)*.

### Correlation of LPC species with BMI and CRP

Since decreased plasma levels of total LPC and LPC species may be due to obesity and the inflammatory status in these subjects, respectively, we correlated BMI and CRP as an indicator of inflammation with total LPC as well as with different LPC species within the group of obese subjects. We found significant negative correlations of BMI with LPC 15∶0, LPC 18∶1, LPC 18∶2, LPC 18∶3, LPC20∶0, and LPC 22∶6. Summing up groups of LPC species, we found significant negative correlations of BMI with all unsaturated, monounsaturated as well as with polyunsaturated LPC. Similar to BMI, CRP correlated negatively with LPC 15∶0, LPC18∶0, LPC 18∶1, LPC18∶2, LPC 20∶0, LPC 20∶4, LPC 22∶5, LPC 22∶6. All analyzed subgroups of LPC species also correlated significantly with CRP. *(* ***Table 2*** *)*.

**Table 2:** Correlation between CRP and BMI with LPC species.

LPC species	CRP		BMI	
	R value	P value	R value	P value
LPC 15:0	**−0.273**	**0.016**	**−0.359**	**0.001**
LPC 16:1	−0.029	0.803	−0.051	0.658
LPC 16:0	−0.183	0.111	−0.032	0.780
LPC 18:3	−0.182	0.114	**−0.251**	**0.028**
LPC 18:2	**−0.434**	**<0.001**	**−0.490**	**<0.001**
LPC 18:1	**−0.419**	**<0.001**	**−0.389**	**<0.001**
LPC 18:0	**−0.305**	**0.007**	−0.054	0.643
LPC 19:0	−0.039	0.733	−0.007	0.951
LPC 20:5	−0.150	0.193	−0.192	0.095
LPC 20:4	**−0.288**	**0.011**	−0.120	0.300
LPC 20:3	−0.044	0.705	−0.027	0.818
LPC 20:0	**−0.403**	**<0.001**	**−0.403**	**<0.001**
LPC 22:6	**−0.357**	**<0.001**	**−0.305**	**0.007**
LPC 22:4	**−0.327**	**0.004**	−0.081	0.483
LPC 22:0	−0.005	0.967	−0.067	0.562
Sum LPC	**−0.328**	**0.004**	−0.190	0.098
Sum LPC saturated	**−0.229**	**0.045**	−0.045	0.697
Sum LPC unsaturated	**−0.431**	**<0.001**	**−0.433**	**<0.001**
Sum LPC monounsaturated	**−0.395**	**<0.001**	**−0.370**	**0.001**
Sum LPC polyunsaturated	**−0.426**	**<0.001**	**−0.442**	**<0.001**

### Correlation of LPC species with BMI during weight loss

We addressed the question if weight loss might be able to normalize LPC plasma concentrations. Therefore, we analyzed the correlation of the ratio of BMI before and after weight loss with the ratio of LPC and LPC species. We found a significant negative correlation of the total LPC ratio with BMI. Among LPC species significant correlations were detected for LPC 16∶0, LPC 18∶1, LPC 20∶0, LPC 20∶4, LPC 22∶5, and LPC 22∶6. Summing up groups of LPC species, we found significant negative correlations of BMI ratio with the ratio of unsaturated, monounsaturated and saturated LPC species *(* ***Table 3*** *)*.

**Table 3:** Correlation between the Ratio t = 1/t = 0 of LPC species with the Ratio t = 1/t = 0 of BMI in obese subjects.

LPC species	BMI	
	R value	P value
LPC 15:0	−0.008	0.954
LPC 16:1	0.002	0.990
LPC 16:0	**−0.401**	**0.002**
LPC 18:3	0.015	0.913
LPC 18:2	−0.183	0.174
LPC 18:1	**−0.293**	**0.027**
LPC 18:0	−0.022	0.873
LPC 19:0	0.255	0.055
LPC 20:5	0.034	0.804
LPC 20:4	**−0.465**	**<0.001**
LPC 20:3	0.044	0.744
LPC 20:0	**−0.313**	**0.018**
LPC 22:6	**−0.303**	**0.022**
LPC 22:4	−0.246	0.065
LPC 22:0	−0.148	0.271
Sum LPC	**−0.310**	**0.019**
Sum LPC saturated	**−0.314**	**0.017**
Sum LPC unsaturated	**−0.261**	**0.050**
Sum LPC monounsaturated	**−0.270**	**0.043**
Sum LPC polyunsaturated	−0.250	0.061

## Discussion

Obesity and related diseases in the context of the metabolic syndrome significantly contribute to major worldwide health problems. Since circulating plasma lipids very likely play an important role in this syndrome complex, we performed quantitative plasma lipid profiling in obesity and weight loss supplementing pre-existing data.

Lipidomic analysis revealed PC, SM, and LPC as most abundant lipid classes in plasma of obese as well as control subjects. Similar results from 21 healthy fasting blood donors have been published by our group previously [24] and are in good accordance with comparative data from others [6].

In comparison to lean controls in our study, plasma LPC levels were found significantly decreased in obese subjects before and still after weight loss. This was due to lower plasma levels of nearly all LPC species, in particular the main species LPC 16∶0, 18∶0, 18∶1 and 18∶2. Similar decreases of most LPC species were found in a mouse model of 6 weeks on a high fat diet [8]. Moreover, in small cohorts (n = 9–11) of lean, obese, and obese individuals with T2D, lower plasma levels of total LPC, LPC 15∶0, 18∶0, 18∶1, 18∶2 and 20∶4 were detected previously [8]. The same tendency was observed for most other LPC species analyzed and could be confirmed by our data from a larger study group. Interestingly, no difference in the LPC profile of obese and obese subjects with type 2 diabetes could be found in that study [8]. In contrast, a study in monozygotic twins revealed in acquired obesity a relationship of obesity with increased LPC levels while ether phospholipids were found decreased [7]. Among the LPC species LPC 16∶0, 18∶0, 18∶1, 18∶2, and 20∶4 were mainly altered in obesity. This study also demonstrated a negative correlation of LPC with insulin sensitivity [7]. In trend, an increase of LPC species towards higher BMI levels was also observed by Graessler et al. comparing the plasma lipidome of men with BMI>27.5 kg/m^2^ relative to a control group of men with BMI <27.5 kg/m^2^. However, significant differences were only detected for LPC 16∶0 [6].

The discrepant data describing alterations in LPC plasma levels related to BMI and obesity remain not fully understood. One important factor may be the exclusion of genetic factors in the twin study by Pietilainen et al. [7] which selectively analyzes the effects of acquired obesity in their study. Genetic factors in obesity, alterations in lipid metabolism and development of the metabolic syndrome are well known (reviewed in [25]) and may contribute significantly to the results of our study and the study of Barber et al. [8]. A recently published study detected a direct correlation of LPC with the number of servings of full-fat dairy foods in overweight and obese subjects with metabolic syndrome [2]. This supports the concept that mainly genetic factors contributing to obesity may be causal for decreased LPC plasma levels rather than nutritive and environmental factors that seem to increase LPC with higher BMI.

In a study published 2008 Schwab et al. [9] could not observe significant alterations in LPC plasma levels after a 33 week period of weight loss in obese subjects. However, we found a significant negative relationship of the total LPC ratio as well as of several LPC ratios with change of BMI ratio during weight loss. This indicates that effective and long-lasting weight loss may indeed be able to normalize altered LPC levels. Studies published so far, were potentially still too short to detect a full revision of profound and long-time lasting alterations in metabolism even after significant weight loss. In addition, decreases in LPC levels may be partially also due to genetic factors contributing to the development of severe overweight.

In our study, total LPC as well as a number of LPC species not only correlate negatively with BMI but also with CRP, possibly indicating a role of LPC as a marker of inflammation in obesity. The association of obesity with elevated levels of CRP, as a marker of inflammation and predictor of cardiovascular risk is well-know (reviewed in [26]) and were also found by others e.g. by Pietilainen et al. [7]. However, the role of LPC in the context of obesity and inflammation still remains poorly understood. A large variety of mainly pro-, but also antiatherogenic effects of LPC as well as a role in acute and chronic inflammation have been described so far [27]. In particular, in chronic inflammatory diseases local and systemic increases are described as characteristic features [28]. Moreover, in cardiovascular disease, certain phospholipases A2 generating LPC from cell membrane or lipoprotein derived PC [27] have been evaluated as biomarkers of cardiovascular risk. This includes the family of secretory phospholipases A2 (sPLA2) of which elevated levels and certain polymorphisms of GIIA sPLA2 predict cardiovascular events. Similar results were described for lipoprotein phospholipase A2 (LpPLA2), indicating higher risk for coronary heart disease and ischemic stroke in individuals with higher LpPLA2 activity or concentration [29].

In contrast, also decreased LPC levels in inflammation and anti-inflammatory effects of LPC have been reported. In sepsis patients absolute concentrations of total LPC as well as of major LPC species were found significantly decreased compared to healthy controls. In these patients the LPC-PC ratio was higher in survivors, compared with non-survivors of sepsis. Low LPC-PC ratios at day 4 and 11, in patients fulfilling all sepsis criteria, were significantly associated with 30 days mortality [30]. These findings were also confirmed in a mouse model of experimental sepsis, in which administration of LPC protected against lethality accompanied by decreased TNFα and IL-1β levels [31]. Interestingly, in cancer patients LPC also correlated negatively with CRP. In this study-population, a decrease of LPC was also associated with cancer-related weight loss [32]. Furthermore, in a recently published study we could demonstrate a beneficial role of the major LPC species LPC 16∶0, 18∶0 and 18∶2 which were negatively associated with total mortality and partially with coronary artery disease [33]. In addition to cardiovascular diseases, a depletion of plasma LPC 18∶2 has recently also been observed in patients with Alzheimeŕs Disease and mild cognitive impairment [34].

Regarding these data, decreased LPC levels in our study population of obese subjects may not only be caused by overweight, but may contribute also to chronic inflammation. LPC effects on inflammatory processes may be mediated, at least in part by LPC binding G protein coupled receptor (GPR) G2A, which appears to play a functional role in the modulation of activation, migration and apoptosis of a variety of immune cells including neutrophils, macrophages and lymphocytes [27], [28].

Our data support a protective and anti-inflammatory and striking role of LPC that is lost in the chronic inflammatory status in obesity. Since obesity is often a life-long process, the study period might have been too short a duration to detect the possible beneficial effect of weight loss.

However, further investigations will be needed to elucidate the pleiotropic role of LPC and other bioactive lipids.
